# Socio-Cognitive Factors Associated With Lifestyle Changes in Response to the COVID-19 Epidemic in the General Population: Results From a Cross-Sectional Study in France

**DOI:** 10.3389/fpsyg.2020.579460

**Published:** 2020-09-29

**Authors:** Aymery Constant, Donaldson Fadael Conserve, Karine Gallopel-Morvan, Jocelyn Raude

**Affiliations:** ^1^EHESP School of Public Health, Rennes, France; ^2^INRAE, INSERM, Univ Rennes, Nutrition Metabolisms and Cancer institute, NuMeCan, Rennes, France; ^3^Milken Institute School of Public Health, George Washington University, Washington, DC, United States

**Keywords:** COVID-19, confinement, physical activity, stress, coping strategics, barriers, eating habits

## Abstract

**Background:**

The aims of the present study were to assess changes in lifestyles in the general population in response to coronavirus disease 2019 (COVID-19) lockdown and the influence of COVID-19 perceptions, as assessed by the Extended Parallel Process Model (EPPM), on these changes.

**Methods:**

Data were collected from 4005 individuals through an online survey conducted 3–4 weeks after the nationwide lockdown implementation in France. Participants were asked whether they practiced five behaviors (i.e., screen watching, snacking, eating fruits and vegetables, exercising, and walking) less often, as often as, or more often than prior to the lockdown. Beliefs and expectations toward the COVID-19 epidemic were also assessed using an adapted version of Witte’s EPPM, together with sociodemographic and environmental variables. Among the respondents consuming regularly alcohol and tobacco, logistic regressions were performed to estimate the Odds ratios (ORs) of increase (yes/no) and decrease (yes/no) in drinking and smoking since the lockdown.

**Results:**

More than 8 in 10 respondents reported unhealthy changes in lifestyle since the lockdown, mostly in relation to physical activity. The unhealthy changes were positively associated with male sex (RR = 1.17; confidence interval [95% CI] = 1.10–1.24), living urban density, having a garden (RR = 1.16 [1.07–1.26]), financial difficulties because of COVID-19 (RR = 1.09 [1.02–1.18]), and lack of fear control (RR = 1.04 [1.01–1.09]) and negatively with cognitive avoidance (RR = 0.92 [0.89–0.95]). Less than 4 in 10 respondents reported healthy changes over the same period, mostly in relation to better eating habits. They were positively associated with living with more than two persons (RR = 1.22 [1.02–1.45]), having a terrace (RR = 1.14 [1.02–1.29], and perceived efficacy (RR = 1.11 [1.04–1.08]) and negatively with being aged 40 or higher. Alcohol consumption overall declined in regular drinkers, while a slight increase in tobacco use was observed in regular smokers.

**Discussion:**

The COVID-19 pandemic and lockdown resulted in frequent and mostly unhealthy changes in lifestyle among the general population. These changes were related to individual and environmental characteristics but also to EPPM appraisals in the wake of fear appeal from COVID-19 campaigns. Communication and preventive measures should include messages and initiatives toward the maintenance of healthy lifestyles during pandemics such as the adaptation of physical activity and eating guidelines to the particular contexts of mobility restriction and infection control.

## Introduction

The coronavirus disease 2019 (COVID-19) pandemic has affected many countries, with more than ten million cases worldwide and more than 500,000 deaths by July 1st 2020 (ECDC, 2020). Several measures were implemented in order to prevent further spread of the disease in the early stages of the pandemic. Nationwide confinement, the restriction of individuals to their homes, was one of the measures enforced in many countries, including France on March 17, 2020 (Augeraud-Veron, 2020). In addition, global and local health authorities used media campaigns to inform about the virus spreads and the number of daily cases and deaths and to promote the recommended actions to prevent infections in mass populations (Lasbeur et al., 2020; Raude et al., 2020). These include regular handwashing, social distancing, avoiding crowed places, and covering mouth and nose, among others.

Though these measures have been vital for preventing the spread of COVID-19, they may have alsoresulted in adverse health effects. Firstly, the ensuing reduction in social (collective training sessions or sport events) and physical (barred access to exercise facilities, or parks)opportunities to exercise may have had a direct effect onsedentary behaviors (Ammar et al., 2020). It must be noted however that each person in France had a 1-hour authorization to exercise locally (Legifrance, 2020). Secondly, prevention campaigns that promoted the recommended actions to prevent infections and intensive press coverage in such a novel and uncertain situation inevitably used fear appeal, which is associated with negative health outcomes, to raise awareness towards the health threat (Tannenbaum et al., 2015). If fear appeal campaigns may motivate adaptive danger control actions such as message acceptance, theymay also generate maladaptive fear control actions such as defensive avoidance or reactance (Witte and Allen, 2000), and unhealthy behaviors in response to chronic stress (Schneiderman et al., 2005; Peters et al., 2013).

According to the extended parallel process model (EPPM), two appraisals typically occur when individuals are exposed to fear appeals (Witte, 1992). The threat appraisal consists of assessing how much threat the fear appeal poses, in terms of severity and susceptibility, while the other assesses perceived efficacy and both self-efficacy (i.e., person’s belief about his/her ability to follow the recommended suggestions successfully) and response efficacy (i.e., beliefs about the effectiveness of the recommended suggestions to avert the threat). When individuals are exposed to a fear appeal, they engage either in danger control, where they cognitively process the message and take action to avoid the threat, or in fear control, where they emotionally repress the message and ignore the threat. A third alternative is to ignore the message, which typically occurs because the threat is perceived as low (Witte, 1994; Maloney et al., 2011; Lewis et al., 2013). The EPPM has advanced understanding of how fear appeals operate, and constitutes a relevant theoretical framework to assess the perceptions of COVID-19 in the general population and why they engage or not in behavioral recommendations.

Health consequences of the COVID-19 pandemic and lockdown are still to be determined comprehensively (Ifdil et al., 2020; Kutlu et al., 2020; Mattioli et al., 2020), but they may concern lifestyles and behaviours (Stanton et al., 2020). Better knowledge of the factors affecting lifestyles amid lockdown may contribute greatly in designing education campaigns and in organizing optimum counseling during and after pandemics. The aims of the present study were to assess changes in lifestyles among the French general population in response to COVID-19 lockdown and the influence of COVID-19 perceptions, as assessed by the EPPM, on these changes.

## Materials and Methods

### Participants and Procedures

Our data was collected through an online survey conducted among 4,005 adults residing in France. The respondents were recruited among 5,000 panelists from the Arcade Research Institute^1^, who agreed to participate regularly to surveys of customer attitudes and experiences in exchange for financial compensation (valid response rate = 80.1%). The objective of the research was to assess the emotional, cognitive, and behavioral response of the French people to the COVID-19 epidemic during the full lockdown. The respondents to this survey were enrolled on the basis of a stratified sampling method to reflect the distribution of the French general population regarding sex, age, occupation, and region. For the present study, we analyzed data from a 2 weeks survey, which were administered 3–4 weeks after the implementation of the lockdown (between 8 and 20 April 2020). The research protocol was registered by the Ecole des Hautes Etudes en Sante Publique (EHESP) School of Public Health Office for Personal Data Protections and approved by the Institutional Review Board of the University Hospital Institute “Mediterranee Infection” (Marseille, France).

### Measures

#### Lifestyle Variable

The dependent variable for the analyses was self-reported change in lifestyle in response to the COVID-19 pandemic in France. Participants were asked whether they practiced five health behaviors (i.e., screen watching, snacking, eating fruits and vegetables, exercising, and walking) less often, as often as, or more often than prior to the lockdown, using first-person questions (e.g., “Since the lockdown, I exercise [less than/as much as/more than] before”). To facilitate the treatment of the behavioral data, responses obtained from these items were added to generate a cumulative score (range 0–5) that enables to measure participants’ positive and negative change in lifestyle behaviors related to the COVID-19 epidemic.

#### Socio-Cognitive Factors

To assess participants’ beliefs and expectations related to the COVID-19 epidemic, we used a range of constructs and variables from the Witte’s EPPM. Items related to these constructs were drawn from the Risk Behavior Diagnosis Scale^2^, adapted to the COVID-19, and translated to French (see Table 4 for the details). This includes perceived susceptibility [3 items, e.g., “It is likely that I will get infected with coronavirus (COVID-19)”] to and severity [3 items, e.g., “I believe that coronavirus infection (COVID-19) is severe”] of the coronavirus infection, fear (3 items, e.g., “The risk of being infected by coronavirus is frightening me”), perceived response efficacy (3 items, e.g., “Measures recommended by the health authorities are effective in preventing coronavirus infection,” and self-efficacy (3 items e.g., “I can easily apply the recommended measures to prevent coronavirus infection”). For each of them, the participants were asked to rate on a Likert-type response scale ranging from 1 (“totally disagree”) to 5 (“totally agree”), and for which the meaning of each value was explicitly indicated.

Sociodemographic and environmental variables were also collected, such as age, gender, level of education, occupational status (active, unemployed, or retired), household income (in euros), size of household, surface area in m^2^, layout (garden, terrace), population density (urban; more than 100,000 inhab; urban; 20,000–100,000 inhab; urban; 2000–20,000 inhab; rural zone), risk factors (alcohol; tobacco; obesity), and financial difficulties (no; yes, unrelated to covid; yes, in relation to covid).

### Data Analysis

Categorical data were expressed as numbers (*N*) and percentages (%), while numerical data were expressed as means ± standard deviations. EPPM factors were estimated using an unweighted least-square factorial analysis, followed by a Promax rotation, a non-orthogonal (oblique) solution in which the factors are allowed to be correlated. This method provides accurate and conservative parameter estimates when using ordinal data (Lee et al., 2012). This item reduction method established which of the 18 items belonged to domains or conceptual areas and which items should be maintained. Items are deleted if they loaded on two or more factors or if they exhibited a correlation coefficient of less than 0.40 with their own factor. Internal consistency reliability was assessed by computing Cronbach’s alpha coefficient (considered satisfactory if higher than or equal to 0.70). Interscale correlations were computed with the non-parametric Spearman’s correlation test. The factors raw scale scores were transformed to a 0–100 scale [((raw score−lowest possible raw score)/possible raw score range) × 100] and compared with one-way analysis of variance. Since our study outcomes were count variables (number of unhealthy/healthy changes in lifestyles since lockdown), we used generalized linear Poisson regression models to estimate the rate ratios (RRs) of changes in lifestyles as a function of sociodemographic variables and factors’ scores of COVID-19, as assessed by the EPPM. Estimates in univariate analysis (model 1) were expressed as rate ratios with 95% confidence intervals (RR [95% CI]). Significant estimates from model 1 were analyzed in a multivariate model (model 2). Among the respondents consuming regularly alcohol and tobacco, logistic regressions were performed to estimate the odds ratios (ORs) of increase (yes/no) and decrease (yes/no) in drinking and smoking since the lockdown. Statistical analyses were performed using the SPSS statistical package, version 19 (SPSS, Chicago, Illinois, United States).

## Results

Of the 4005 individuals who completed the survey (Table 1), a majority were women (55.4%) professionally active (41.8%) or retired (21.6%) with a net income above 3000 euros per month (53.8%). Most of them lived in urban environments (75.9%), in multi-person households (78.0%), with a living area of 80 m^2^ or more (78.0%) with a garden (66.3%) or/and a terrace (58.2%). A majority of respondents were alcohol drinkers (60.1%) while 26.5% were smokers and 3.9% had obesity. More than 1 in 5 participants (22.7%) reported financial difficulties related to lockdown. More than 8 in 10 respondents reported unhealthy changes in lifestyle since the lockdown (Table 2), while less than 4 in 10 reported healthy changes. Although 4.3% of the participants reported that they tested positive for COVID-19, there were no significant difference between the non-infected and infected participants regarding lifestyles changes under study. The most frequently reported unhealthy changes (Table 3) were decreased walking (60.0%) and exercising (45.4%) and increased screen watching (59.0%). The most frequently reported healthy changes were decreased snacking (18.2%), increased FV consumption (13.1%), and exercising (11.3%).

**TABLE 1:** Participants’ characteristics (*N* = 4005).

Variables		*N* (%)
Female sex		2051 (51.2)
Age in years	60 and older	1032 (25.8)
	40–59	1484 (37.1)
	18–39	1489 (37.2)
Professional status	Active	2715 (67.8)
	Retired	867 (21.6)
	Unemployed	423 (10.6)
Net income (in euros)	More than 4000	1150 (28.7)
	2000–3999	1758 (43.9)
	1500–1999	550 (13.7)
	<1500	547 (13.7)
People in the household	Three or more	1644 (41.0)
	Two	1479 (36.9)
	One	882 (22.0)
Surface area in m^2^	100 and more	1644 (41.0)
	80–100	1564 (39.1)
	<80	882 (22.0)
Layout	Garden	2657 (66.3)
	Terrace	2330 (58.2)
Population density	Urban; more than 100,000	750 (18.7)
	Urban; 20,000–100,000	1049 (26.2)
	Urban; 2000–20,000	1241 (31.0)
	Rural zone	965 (24.1)
Risk factors	Alcohol use	2409 (60.1)
	Tobacco use	1062 (26.5)
	Obesity	158 (3.9)
Financial difficulties	Yes, related to covid	894 (22.3)
	Yes, unrelated to covid	700 (17.5)
	None	2411 (60.2)

**TABLE 2:** Number of healthy and unhealthy changes in lifestyle since lockdown (*N* = 4005).

	Type of behavior change	
	
	Healthy	Unhealthy
Number	*N* (%)	*N* (%)
0	2453 (61.2)	649 (16.8)
1	1069 (26.7)	795 (19.9)
2	376 (9.4)	1068 (26.7)
3	89 (2.2)	937 (23.4)
4	17 (0.4)	455 (11.4)
5	1 (0)	101 (2.5)

**TABLE 3:** Participants’ reported changes in lifestyle amid lockdown (*N* = 4005).

	Change since lockdown		
	
	Decrease	Unchanged	Increase
Behaviors	*N* (%)	*N* (%)	*N* (%)
Screen watching	145 (3.6)	1498 (37.4)	2362 (59.0)
Snacking	727 (18.2)	2318 (57.9)	960 (24.0)
Eating fruits and vegetables	525 (13.1)	2957 (73.8)	523 (13.1)
Exercise	1818 (45.4)	1736 (43.3)	451 (11.3)
Walking	2402 (60.0)	1288 (32.2)	315 (7.8)

**TABLE 4:** Factor matrix: items and factor loadings for the five-factor solution of the extended parallel process model applied to COVID-19 (*N* = 2121).

	Factors				
	
Items	Efficacy	Lack of fear control	Severity	Vulnerability	Avoidance
Measures recommended by authorities are effective in preventing the COVID-19	0.606				
Actions recommended by scientists work in preventing the COVID-19	0.686				
If I follow expert advices, I am less likely to get the COVID-19	0.680				
I am able to follow authorities recommendations to prevent getting the COVID-19	0.767				
I have the skills/time/money to apply recommended measures to prevent COVID-19	0.712				
I can easily apply the recommended measures to prevent COVID-19	0.809				
The risk of being infected worries me particularly		0.907			
The risk of being infected is frightening me		0.882			
The risk of being infected make me nervous		0.848			
When I go for a walk, I always keep in mind that I can be infected		0.529			
I believe that COVID-19 is severe			0.874		
I believe that COVID-19 has serious negative consequences for health			0.766		
I believe that COVID-19 is extremely harmful			0.872		
It is likely that I will get the COVID-19 in the next weeks				0.896	
I am at risk for getting the COVID-19				0.370	
It is possible that I will get the COVID-19 in the next weeks				0.861	
When I go shopping, I tend to avoid thinking about the risk of being infected					0.703
When I come across others people outside, I tend to avoid thinking about the risk of being infected					0.887
Eigenvalue	4.97	3.30	1.78	1.46	1.14
% of explained variance	27.6	18.3	9.9	8.1	6.3
Cronbach’s alpha	0.86	0.88	0.88	0.74	0.77

Of the 4005 respondents, 2121 (52.9%) completed the EPPM items without missing values. Respondents particularly discarded items with vague wording (e.g., “I am at risk for getting the COVID-19”) in a context where scientific knowledge on COVID-19 varied from day to day. Unweighted least-square exploratory factorial analysis, followed by a Promax rotation, was performed on the 18 items (Table 4). Eigenvalues for the first six factors were 4.97, 3.30, 1.78, 1.46, 1.14, and 0.81, suggesting a five-factor solution explaining 69.9% of the common variance of the data. Factor #1 included six items related to response efficacy and self-efficacy and was interpreted as expressing efficacy; Factor #2 included four items related to lack of fear control; Factor #3 included three items expressing perceived COVID-19 severity; Factor #4 included three items expressing perceived vulnerability to COVID-19; and Factor #5 included items related to cognitive avoidance. Factors showed satisfactory internal validity (Cronbach’s coefficient > 0.70). Interscale correlations between scores of severity, vulnerability, and lack of fear control (Table 5) were low to moderate (ranging from 0.19 to 0.58), showing that these factors were related but distinct. On the other hand, correlations between avoidance and others factors were low to absent (ranging from 0.07 to 0.10), suggesting that cognitive avoidance was the standalone coping strategy. The raw scale scores were transformed to a 0–100 scale [((raw score−lowest possible raw score)/possible raw score range) × 100]. Higher T-scores in the respective scales are indicative of greater perceived efficacy, lack of fear control, severity, vulnerability, or avoidance. Efficacy (81.9 ± 13.2) and severity (82.7 ± 16.6) reached highest scores on a 100-point scale, followed by lack of fear control (69.4 ± 19.5), perceived vulnerability (63.1 ± 21.5), and avoidance (59.7 ± 18.9). Differences between T-scores were significant, except for efficacy and severity.

**TABLE 5:** Spearman correlations between factors of the extended parallel process model applied to COVID-19 (*N* = 2121).

	Efficacy	Lack of fear control	Severity	Vulnerability	Avoidance
Efficacy	1				
Lack of fear control	0.13	1			
Severity	0.34	0.58	1		
Vulnerability	−0.06	0.35	0.19	1	
Avoidance	0.09	0.07	0.08	0.10	1

Estimate of unhealthy changes in univariate analysis (Table 6) increased with male sex, COVID-19-induced financial difficulties, having a garden or terrace, living in urban environments, obesity, and level of fear, perceived severity, and vulnerability and decreased with age older than 60 years, surface area, and level of avoidance. In multivariate analyses, estimates of unhealthy changes increased with male sex, COVID-19-induced financial difficulties, having a garden, living in urban environments, and elevated level of fear and decreased with age older than 60 years, surface area, and level of avoidance.

**TABLE 6:** Rate ratios and 95% confidence intervals (RR [95% CI]) of the number of unhealthy changes in lifestyle since lockdown (*N* = 2196); Poisson regression.

		Univariate	Multivariate
Variables		RR [95% CI]	RR [95% CI]
Male sex		**1.19 [1.12–1.26]**	**1.17 [1.10–1.24]**
Age in years	60 and older	**0.99 [0.84–0.99]**	0.94 [0.86–1.03]
	40–59	1.04 [0.97–1.11]	1.03 [0.96–1.10]
	18–39	1	1
Professional status	Active	1.03 [0.93–1.15]	
	Retired	0.93 [0.82–1.05]	
	Unemployed	1	
Net income (in euros)	More than 4000	1.02 [0.92–1.12]	
	2000–3999	0.94 [0.86–1.04]	
	1500–1999	1.01 [0.90–1.14]	
	< 1500	1	
Financial difficulties	Yes, related to covid	**1.13 [1.06–1.22]**	**1.09 [1.02–1.18]**
	Yes, unrelated to covid	0.98 [0.90–1.074]	0.96 [0.87–1.05]
	None	1	1
Number of household bers	Three or more	0.95 [0.88–1.03]	
	Two	0.93 [0.86–1.01]	
	One	1	
Surface area in m^2^	100 and more	**0.85 [0.78–0.91]**	0.95 [0.87–1.03]
	80–100	**0.90 [0.83–0.97]**	0.95 [0.87–1.05]
	<80	1	1
Layout	Garden	**1.23 [1.15–1.30]**	**1.16 [1.07–1.26]**
	Terrace	**1.10 [1.04–1.17]**	0.97 [0.90–1.05]
Population density	Urban; more than 100000	**1.30 [1.19–1.42]**	**1.18 [1.07–1.31]**
	Urban; 20 000–100 000	**1.20 [1.10–1.31]**	**1.11 [1.01–1.22]**
	Urban; 2000–20000	**1.14 [1.05–1.24]**	**1.10 [1.01–1.20]**
	Rural zone	1	1
Risk factors (yes/no)	Obesity	**1.16 [1.01–1.34]**	1.13 [0.98–1.31]
	Alcohol	0.96 [0.91–1.02]	
	Tobacco	0.95 [0.89–1.02]	
Factor score	F1: efficacy	1.00 [0.97–1.03]	
	F2: lack of fear control	**1.09 [1.06–1.12]**	**1.04 [1.01–1.09]**
	F3: severity	**1.06 [1.03–1.10]**	1.02 [0.97–1.06]
	F4: vulnerability	**1.05 [1.02–1.09]**	1.03 [0.99–1.06]
	F5: avoidance	**0.92 [0.89–0.95]**	**0.92 [0.89–0.95]**

**Significant results are marked in bold.**

Estimates of healthy changes in univariate analysis (Table 7) increased with COVID-19-induced financial difficulties, living in a household with 3 or more persons, having a terrace, and levels of adherence, fear, perceived severity, and avoidance and decreased with age and income between 2000 and 3999 euros per month. In multivariate analyses, estimates of healthy changes increased with living in a household with 3 or more persons, having a terrace, and levels of adherence and decreased with older age.

**TABLE 7:** Rate ratios and 95% confidence intervals (RR [95% CI]) of the number of healthy change in lifestyle since lockdown (*N* = 2196); Poisson regression.

		Univariate	Multivariate
Variables		RR [95% CI]	RR [95% CI]
Male sex		1.05 [0.94–1.17]	
Age in years	60 and older	**0.74 [0.63–0.87]**	**0.77 [0.65–0.92]**
	40–59	**0.80 [0.71–0.90]**	**0.80 [0.71–0.91]**
	18–39	1	1
Professional status	Active	1.08 [0.90–1.31]	
	Retired	0.91 [0.72–1.15]	
	Unemployed	1	
Net income (in euros)	More than 4000	0.91 [0.76–1.09]	0.92 [0.75–1.13]
	2000–3999	**0.84 [0.71–0.99]**	0.83 [0.69–1.01]
	1500–1999	0.96 [0.78–1.18]	0.98 [0.80–1.21]
	< 1500	1	
Financial difficulties	Yes, related to covid	**1.20 [1.05–1.36]**	1.12 [0.97–1.28]
	Yes, unrelated to covid	0.90 [0.75–1.05]	0.90 [0.75–1.08]
	None	1	
Number of household bers	Three or more	**1.17 [1.01–1.36]**	**1.22 [1.02–1.45]**
	Two	1.09 [0.93–1.28]	1.19 [1.00–1.42]
	One	1	1
Surface area in m^2^	100 and more	0.98 [0.87–1.11]	
	80–100	0.97 [0.83–1.13]	
	< 80	1	
Layout	Garden	1.01 [0.90–1.14]	
	Terrace	**1.13 [1.01–1.26]**	**1.14 [1.02–1.29]**
Population density	Urban; more than 100000	1.15 [0.97–1.36]	
	Urban; 20 000–100 000	1.07 [0.92–1.25]	
	Urban; 2000–20000	0.98 [0.77–1.05]	
	Rural zone	1	
Risk factors (yes/no)	Obesity	1.11 [0.85–1.45]	
	Alcohol	0.91 [0.82–1.02]	
	Tobacco	1.12 [0.99–1.26]	1.07 [0.95–1.21]
Factor scores	F1: efficacy	**1.12 [1.06–1.20]**	**1.11 [1.04–1.08**]
	F2: lack of fear control	**1.06 [1.01–1.13]**	1.00 [0.92–1.08]
	F3: severity	**1.08 [1.02–1.15]**	1.07 [0.98–1.16]
	F4: vulnerability	0.95 [0.90–1.01]	
	F5: avoidance	**1.05 [0.99–1.12]**	1.03 [0.97–1.10]

**Significant results are marked in bold.**

### Alcohol and Tobacco Consumption

Of the 2409 regular drinkers, 356 (14.8%) increased and 508 (21.1%) decreased alcohol consumption since the lockdown. In multivariate analyses, estimates of higher drinking increased with having a terrace (OR = 1.76 [1.14–2.71)] and decreased with age (60 years or older: OR = 0.56 [0.34–0.91], 40–59 years: OR = 0.61 [0.43–0.87]; reference: 18–39 years). Estimates of lower drinking increased with living in dense urban area (> 100 000 inhabitants) as compared to rural areas (OR = 1.66 [1.07–2.58]), and level of efficacy (OR = 1.27 [1.09–1.48]), and decreased with age 40–59 years (OR = 0.64 [0.47–0.88)]. Of the 1062 regular smokers, 231 (21.8%) increased and 177 (16.7%) decreased their tobacco consumption. These changes were unrelated to factors under study.

## Discussion

More than 8 in 10 respondents reported unhealthy changes in lifestyle since the lockdown, mostly in relation to unhealthy changes in lifestyles which were common amid COVID-19 confinement, affecting especially physical activity. The unhealthy changes were positively associated with male sex, living in dense urban areas, having a garden, financial difficulties because of COVID-19, and lack of fear control and negatively associated with cognitive avoidance. Less than 4 in 10 respondents reported healthy changes over the same period, mostly in relation to better eating habits. They were positively associated with associated with living with more than two persons, having a terrace, and perceived efficacy and were negatively related to being aged 40 or higher.

The implementation of confinement and physical distancing, including mobility restrictions, banning of mass gatherings, closure of schools and work activities, isolation, and quarantine, helped control the first wave of the COVID-19 pandemic but resulted in overall unhealthy changes in lifestyle in France and elsewhere (Stanton et al., 2020). Expectedly, a majority of respondents reported increased sedentary and decreased walking in response to lockdown. As four times more respondents reported decreased than increased exercising, the 1 h permission daily given to go out on exercise failed to compensate for mobility restriction. It must be noted that grounds for sport activities were also shut down, making impossible all forms of exercise besides walking and running. The picture is more mitigated when it comes to eating habits, since healthy changes nearly compensated unhealthy changes.

According to EPPM, if people assume that they are strongly exposed to a disease (threat appraisal), the efficacy appraisal of coping strategies will change their attitudes and behaviors. In the present study, factor analysis revealed a five-factor structure underlying perceptions about the COVID-19. Response efficacy and self-efficacy formed together the “perceived efficacy appraisal,” which reached a high score in our study sample, indicating a sound adherence to recommended preventive measures. Respondents also reported high scores of severity, showing they were well aware of the seriousness of the COVID-19 consequences amid communication campaigns. This perception theoretically forms the “threat appraisal” together with vulnerability in the EPPM but correlated more to the “lack of fear control” score possibly due to respondents’ strong reactions to the fear appeal communication about COVID-19, without necessarily considering themselves as highly vulnerable. Altogether, response efficacy seemed to equal the threat appraisal, which indicates a “danger control” process, in which individuals are motivated to take action to lessen the threat. Nevertheless, cognitive avoidance was reported as a standalone coping strategy to provide some distance from the steady stream of information about COVID-19 (Park et al., 2020).

The relationships observed between severity, vulnerability and unhealthy changes in the univariate analysis becomes non-significant when entered together with lack of fear control in the multivariate model. This finding indicates that threat appraisal may negatively influence changes in lifestyles through uncontrolled fear responses, precluding going out for exercise/walking or prompting emotional overeating. In addition, unhealthy changes were higher among men and respondents with financial difficulties – which may indicate a maladaptive response to stress (Park et al., 2020; Verma and Mishra, 2020). Previous research has reported that fear appeal campaigns are effective when the communication depicts relatively high amounts of fear, stresses severity, and susceptibility of the threat, and recommends one-time only behaviors and includes an efficacy message (Tannenbaum et al., 2015). However, in a context of public health and economic uncertainty such as the early stages of the COVID-19 crisis, fear appeal may also induce negative side effects among vulnerable individuals. In contrast to other studies (Soga et al., 2017; Saltzman et al., 2020), living in densely populated areas and having a garden had negative effects on health behaviors. In the context of COVID-19 epidemic and mobility restrictions, rural residents possibly had greater access to outdoor exercise areas than urban dwellers, while garden owners could enjoy open air without much effort, which fostered sedentary. Conversely, avoiding thinking about the COVID-19 (Umucu and Lee, 2020) somewhat limited unhealthy changes in lifestyles, probably by reducing the fear to go out.

A minority of respondents took advantage of the lockdown to improve their habits, especially toward food. The relationships observed between severity, fear control, and healthy changes in the univariate analysis become non-significant when entered together with efficacy in the multivariate model. This could indicate that some respondents in “danger control appraisal” (high efficacy, high threat) improved their behaviors in response to the COVID-19, when obesity was considered a risk factor for disease mortality. Healthy changes were also related to the size of household, may be in relation to increased family and social support (Hempler et al., 2016; Saltzman et al., 2020) and to the need to protect close relatives. On the other hand, these positive evolutions were less frequent in respondents aged 40 and older for unclear reasons, although aging often entails the need to make changes in lifestyle (US National Research Council Committee on Aging Frontiers in Social Psychology, Personality, and Adult Developmental Psychology, 2006).

Finally, analyses conducted among regular drinkers revealed that alcohol consumption overall declined in regular drinkers. One possible explanation is that the lockdown precluded most – if not all – social activities, which often involve alcohol in France (INSERM Collective Expert Reports [Internet], 2003). Higher drinking decreased with age, which is in line with previous findings (Chodkiewicz et al., 2020), probably because the threat of contracting COVID-19 might have motivated vulnerable populations to minimize adverse health outcomes. However, being aged 40–59 years was also associated with higher drinking, which may reflect the high rate of alcohol problem in this age group (Constant et al., 2017). Conversely, a slight increase in tobacco use was observed in regular smokers, showing that stress related to the COVID-19 pandemic affected risk behaviors in different ways (Bommele et al., 2020). While the threat of contracting COVID-19 might have motivated a minority of regular smokers to improve their health, boredom, and restrictions in movement might have fostered smoking in others.

This study must be interpreted in light of its limitations. Firstly, the assessment of lifestyle changes in this study was suboptimal, since it was based on individual recall methods, without reproducibility assessment. Secondly, the cross-sectional design does not allow causal inferences about relationships between variables to be determined. Furthermore, missing data precluded the investigation of EPPM appraisal in the total study sample. Thirdly, personality variables such as anxiety trait and pessimism may have a pivotal influence on appraisals and were not assessed. Finally, data were collected in a cohort including a minority of individuals with deprived socioeconomic backgrounds, which may limit the generalizability of our results. Since the monitoring of lockdown adverse effects is suboptimal, the large size of our cohort and the inclusion of diverse professions and socioeconomic groups nevertheless have offered an interesting opportunity to assess threat appraisals and behavioral changes amid the COVID-19 pandemic in the general population.

Our findings suggest that the coronavirus disease pandemic and lockdown resulted in frequent and mostly unhealthy changes in lifestyle in the general population. These changes were related to individual and environmental characteristics but also to EPPM appraisals in the wake of fear appeal COVID-19 campaigns. Communication and preventive measures should include messages and initiatives toward the maintenance of healthy lifestyles during pandemics. This goes through the adaptation of physical activity and eating guidelines to the particular contexts of mobility restriction and infection control.

## Data Availability Statement

The raw data supporting the conclusions of this article will be made available by the authors, without undue reservation.

## Ethics Statement

The studies involving human participants were reviewed and approved by the Institutional Review Board of the University Hospital Institute “Mediterranee Infection” (Marseille, France). The patients/participants provided their written informed consent to participate in this study.

## Author Contributions

AC, DC, KG-M, and JR contributed to the conception and design of the study and interpreted the data and drafted the final manuscript. KG-M suggested the theoretical framework. AC performed the statistical analysis and wrote the first draft of the manuscript. All authors read and approved the manuscript.

## Conflict of Interest

The authors declare that the research was conducted in the absence of any commercial or financial relationships that could be construed as a potential conflict of interest.

## Footnotes

1 www.panelia.fr

2 https://msu.edu/∼wittek/rbd.htm
